# Resting metabolic rate is increased after a series of whole body vibration in young men

**DOI:** 10.1038/s41598-023-44543-3

**Published:** 2023-10-11

**Authors:** Marcin Maciejczyk, Marek Bawelski, Magdalena Wiecek, Tomasz Palka, Przemyslaw Bujas, Anna Piotrowska, Zbigniew Szygula

**Affiliations:** 1 Department of Physiology and Biochemistry, University of Physical Education, Kraków, Poland; 2 Department of Theory of Sport and Kinesiology, University of Physical Education, Kraków, Poland; 3 Department of Chemistry and Biochemistry, University of Physical Education, Kraków, Poland; 4 Department of Nutrition and Sport Medicine, University of Physical Education, Kraków, Poland

**Keywords:** Biological techniques, Biophysics, Physiology, Environmental sciences, Environmental social sciences, Anatomy, Biomarkers, Health care, Health occupations, Medical research

## Abstract

Resting metabolic rate (RMR) is the largest component of total energy expenditure and increasing it can be of great importance in reducing excess body fatness. Whole body vibration (WBV) can affect energy expenditure during single session of WBV, but the effects of repeated WBV on resting metabolic rate have not been reported. The purpose of this study was to investigate whether a series of WBV would increase resting metabolism in young men. Thirty-two healthy men aged of 21–23 were recruited and randomly assigned to two 16-member groups: a group participating in the WBV intervention and a group without the intervention. The intervention lasted 2 weeks and WBV was performed 5 times a week. Diet, physical activity, body composition and resting metabolic rate were analyzed in the participants. In WBV group significantly increased resting oxygen uptake (*p* = 0.049) and consequently RMR (*p* = 0.035) after the intervention. Similar changes were not observed in the control group. This indicates that applied type of vibration in this study can be an addition to obesity therapy, in which, WBV can, among other beneficial metabolic effects, increase RMR and thus total energy expenditure.

## Introduction

Resting metabolic rate (RMR) is defined as the body’s energy expenditure at rest, when a person is awake, in a post-absorptive, thermoneutral state, and has not exercised for typically 12 h^1,2^. Resting metabolic rate is the largest component of total energy expenditure^3^ and increasing it can be of great importance, for example, in reducing excess body fatness. One of the ways to increase RMR is increasing physical activity, which, while leading to increased muscle mass (resistance training), also increases RMR^4^. In recent years, there has been a significant increase in obesity and obesity-related diseases, and this trend will continue to grow^5^. In order to treat obesity or prevent it, it seems necessary to look for methods that increase daily total energy expenditure. A promising direction of research may be the use of whole-body vibration (WBV) for this purpose. WBV has many positive metabolic effects, that can be used both in therapies for various diseases as well as in sports^6,7^. These include such WBV effects as improving blood circulation (blood flow)^8^, improving muscle strength and bone density^9^, and improving nervous system function^10^. For this reason, WBV is used in the treatment or prevention of many diseases, e.g. COVID-19 and quality of life^11^, osteoporosis^12^, chronic low back pain^13^, sarcopenia^14^ or ataxia^15^. WBV can also affect energy expenditure. Milanese et al.^16^ indicted that WBV can significantly increase the metabolic cost of exercise. RMR was also shown to increase during a single session of WBV in the supine position, but the observed effect did not persist after WBV ended^17^. The increased oxygen uptake during WBV was also confirmed^18^. To date, it has not been reported whether the observed metabolic effects of WBV may be permanent, i.e., induced by repeated exposure to vibration stimulation. We therefore hypothesized that systematically repeated WBV may induce an increase in resting metabolic rate, contributing to an increase in total energy expenditure. The purpose of this study was to investigate whether a series of WBV would increase resting metabolism in young men.

## Methods

### Study design

The study was a parallel randomized trial. Thirty-two healthy men between the aged 21 to 23 years old were recruited and randomly assigned to two 16-member groups: a group participating in the WBV intervention (n = 16) and a group without the intervention (placebo: PL, n = 16). Participants drew numbers from 1 to 32: even numbers were placed in the WBV group, odd numbers in the PL group. The men were physically active, but did not engage in competitive sports. Only healthy men with normal BMI and similar physical activity were included in the study. Exclusion criteria were metabolic diseases, overweight or obesity, use of stimulants and pharmaceuticals, and anxiety disorders. Medical qualification for participation in the project was made by a physician.

Diet, physical activity, body composition and resting metabolic rate were analyzed in the participants. Body composition analysis and RMR measurement were performed twice, i.e. before and after completion of the WBV series (n = 10). The intervention lasted 2 weeks and WBV was performed 5 times a week (Monday–Friday) in the hours up to noon. All subjects completed the full series of 10 WBV, as well as all scheduled tests were performed in the study. Participation in the study was voluntary, and participants at any stage of the project were allowed to opt out of further participation. All study participants signed a consent form to participate in the project. The project was approved by the Bioethics Committee of Opole Medical School (no. KB/56/NOZ/2019).

### Whole body vibration

Whole body vibration was performed in the vibration therapy laboratory, in which constant environmental conditions were maintained. The average temperature in the laboratory was 21.7 ± 0.46 °C and the humidity was 54.2 ± 0.70%. Cycloidal-oscillatory vibration was used in the study. WBV was applied by a physiotherapist/exercise physiologist, and during WBV the participant was supervised by the researcher. The intervention was performed in a prone position, as previously described^17^, with the use of a RAM Vitberg + Base Module (active medical device, class IIa) enhanced with a RAM Vitberg + Metabolism module (active medical device, class I) (Vitberg, Poland). Whole body vibration was applied using the Base Module, applying vibration to the trunk, upper limbs and thighs area, and additionally local vibration (Metabolism module) directed to the abdominal area. The physical stimulus was continuous vibration with variable values of frequency, amplitude and acceleration, which ranged, respectively: 25–52 Hz, 0.1–0.5 mm and 6.9–13.5 m/s^2^. The vibration stimulus was generated in three directional perpendicular (3D). A single WBV lasted 29 min. During the placebo treatment, the similar device was used, which did not generate vibration, but only a sound identical to the vibration. The procedures and body position were identical to the vibration intervention.

### Body height, body mass and body composition

The body composition of the men was examined twice before and after the intervention. Body composition was measured by Dual Energy X-ray Absorptiometry (DEXA) (Lunar Prodigy, GE, USA) according to the manufacturer’s guidelines. Body mass (BM), fat-free mass (FFM), fat mass (FM) and percent body fat (%FAT) were determined. Body height was measured without shoes, in a standing position to the nearest 1 mm, with the head in the Frankfurt plane, using a stadiometer (Seca, Germany). In addition, body mass index (BMI) was calculated for each subject.

### Diet and physical activity

Men completed 4-day food diaries, in which they recorded the weight or volume of each food consumed. The serving size was assessed subjectively by the participant on the basis of the Album of Product and Food Photography^19^. The caloric content of the diet and the proportion of carbohydrates, proteins and fats in the diet was then calculated by a qualified nutritionist, using the Diet 6.0 software (Food and Nutrition Institute, Warsaw, Poland).

Physical activity was assessed using the International Physical Activity Questionnaire (IPAQ, short Polish version^20^), which the participants completed on the first day of the intervention. Before completing the questionnaire, they were instructed on its purpose and how to fill it out. The questionnaire was completed in the presence of the researcher, who instructed or answered any questions the respondents might have.

### Resting metabolic rate

RMR was measured in fasting, always at the same time of day (morning), between 6:00 a.m. and 10:00 a.m. Prior to the first RMR measurement, participants were instructed on how to prepare for the measurement, i.e. avoid exercise for 3 days before the scheduled measurement, be properly hydrated, and not use any stimulants before the study (nicotine, caffeine). Participants were also recommended not to change their diet or physical activity during the intervention. RMR was measured in the supine position in an air-conditioned laboratory, at a constant temperature of 21 °C, after a prior rest of about 15 min in the supine position. Resting metabolic rate, was measured by indirect calorimetry using a Cortex MetaLyzer 3R ergospirometer (Germany), using the breath by breath method. The ergospirometer was calibrated each time according to the manufacturer’s requirements (gas and volume calibration). Oxygen uptake (VO_2_), carbon dioxide production (VCO_2_), respiratory quotient (RQ), RMR, and substrate utilization (carbohydrate [CHO], fat [FAT], protein [PRO]) and energy expenditure (EE) from each substrate were measured during the measurement. Resting metabolism was expressed absolutely (kcal/day) and relatively to body mass (kcal/kg/day) and to body surface area (RMR/BSA) (kg/m^2^/day). RMR and other indices measured by calorimetry were determined from a 5-min measurement period during which steady state was observed in oxygen uptake. The steady state was considered to be fluctuations in oxygen uptake within ± 10%. The averages from steady state were used to calculate REE using the Weir^21^ formula without using urinary urea nitrogen. All calculations were performed using dedicated metabolic rate measurement software provided by ergospirometer manufacturer (Cortex, Germany).

### Statistical analysis

Sample size was estimated a priori using G*Power 3.1.9.7 (Dusseldorf, Germany). The following options were selected in the software: test family = f tests; statistical test = ANOVA repeated measures, within-between interaction; type of power analysis = compute required sample size—given α, power, and effect size. Input parameters into the software were as follows: effect size f = 0.4; α error probability = 0.01; power = 0.95; number of groups and measurements = 2; correlation among measures = 0.5; nonsphericity correction = 1.0. The required sample size was 16 subjects per group (total sample size = 32). Data distribution was checked using the Shapiro–Wilk test. Homogeneity of variance within the groups was tested via Levene’s test Analysis of variance (ANOVA) with repeated measures or one-way ANOVA was used to analyze the data obtained. In case of significant changes (*p* < 0.05) in the analysis of variance, post-hoc analysis was performed using the Tukey test. Additionaly, in post hoc analysis (if significant), the effect size (Cohen’s d) between baseline and WBV (placebo) treatment was calculated and interpreted as small (0.20), medium (0.50), or large (0.80)^22^. Data are presented as mean and standard deviation. The STATISTICA 13 package (StatSoft, Inc., Tulsa, OK, USA) was used for calculations.

### Ethics declaration

The study was conducted in accordance with the Declaration of Helsinki and approved by the Bioethics Committee of Opole Medical School in Poland (no. KB/56/NOZ/2019). All participants were informed about the study protocol, voluntarily took part in the experiment and signed informed consent.

## Results

No adverse effects were reported during the intervention.

### Physical activity and diet

The groups did not differ significantly (f = 0.46, *p* = 0.50) in physical activity level, which averaged 4156 ± 942 MET-minutes/week in the WBV group and 3902 ± 1163 MET-minutes/week in the PL group. There were also no significant differences in the caloric content of the participants’ diet (f = 0.49, *p* = 0.48). The average dietary energy supply in the WBV group was 3036.5 ± 788.3 kcal/day and in the PL group 2829.6 ± 870.1 kcal/day. Both groups had similar proportions of carbohydrates (378.0 ± 126.8 g (WBV) vs. 328.7 ± 95.4 g (PL); f = 1.54, *p* = 0.22), proteins (respectively: 133.4 ± 31.4 g vs. 128.6 ± 44.4; f = 0.12, *p* = 0.72) and fats (respectively: 112.8 ± 30.2 g vs. 112.2 ± 54.4 g; f = 0.001, *p* = 0.97) in the diet.

### Somatic build

There were no significant differences in body height, body mass or body composition between WBV and PL group. The parameters did not change significantly during the intervention either (Table 1).

**Table 1:** Age, body height and mass and body composition of study participants before and after the intervention.

Variable	Group	Baseline	Post	Effect: group F (*p*)	Effect: time F (*p*)	Interaction F (*p*)	Time change: pre vs. post *p*
Age (years)	WBV	20.91 ± 1.07	–	23.94 (< 0.001)	–	–	–
	PL	22.92 ± 1.24	–				–
BH (cm)	WBV	176.53 ± 6.63	–	1.88 (0.18)	–	–	–
	PL	180.18 ± 8.34	–				–
BM (kg)	WBV	74.93 ± 6.39	74.62 ± 6.17	0.18 (0.66)	0.33 (0.56)	2.474 (0.12)	0.48
	PL	75.95 ± 9.59	76.10 ± 9.68				0.13
BMI	WBV	24.07 ± 1.96	23.97 ± 1.87	0.75 (0.39)	0.008 (0.92)	1.78 (0.19)	0.38
	PL	23.35 ± 2.15	23.44 ± 2.17				0.32
FFM (kg)	WBV	61.19 ± 5.43	61.27 ± 5.45	0.25 (0.62)	2.11 (0.15)	0.670 (0.41)	0.11
	PL	62.32 ± 8.14	62.60 ± 8.17				0.65
FAT (kg)	WBV	12.94 ± 4.06	12.96 ± 4.03	0.14 (0.71)	1.00 (0.32)	1.66 (0.20)	0.11
	PL	13.63 ± 4.96	13.48 ± 4.87				0.84
FAT (%)	WBV	18.07 ± 5.19	18.09 ± 5.16	0.04 (0.82)	1.70 (0.20)	2.40 (0.13)	0.52
	PL	17.79 ± 5.29	17.57 ± 5.12				0.86

*BH* body height, *BM* body mass, *BMI* body mass index, *FFM* fat free mass, *WBV* whole-body vibration, *PL* placebo.

### Resting metabolic rate

There were no significant intergroup differences in the level of the parameters studied (Table 2). However, significant changes were observed between measurements. In the WBV group significantly increased resting oxygen uptake (*p* = 0.049) and consequently RMR (*p* = 0.035) after intervention. The significant increase in RMR in the WBV group was due to increased resting carbohydrate utilization i.e. 330.88 ± 98.1 g/day (baseline) vs. 407.13 ± 111.9 g/day (post intervention) (*p* = 0.024). Similar changes were not observed in the PL group (Table 2).

**Table 2:** Effects of whole-body vibration series on resting metabolic rate in participants.

Variable	Group	Baseline	Post	Effect: group F (*p*)	Effect: time F (*p*)	Interaction F (*p*)	Time change: pre vs. post *p*
VO_2_ (L/min)	WBV	0.30 ± 0.04	0.33 ± 0.06	3.40 (0.07)	0.01 (0.91)	7.56 (0.01)	0.049
	PL	0.37 ± 0.05	0.34 ± 0.09				0.076
VCO_2_ (L/min)	WBV	0.27 ± 0.04	0.30 ± 0.06	3.71 (0.06)	3.62 (0.06)	4.97 (0.03)	0.005
	PL	0.32 ± 0.06	0.32 ± 0.07				0.821
RQ	WBV	0.89 ± 0.05	0.92 ± 0.05	0.40 (0.53)	7.71 (0.0)	1.92 (0.1)	0.325
	PL	0.88 ± 0.09	0.96 ± 0.13				0.007
RMR (kcal/day)	WBV	2124.19 ± 274.4	2330.88 ± 430.8	3.19 (0.08)	0.19 (0.66)	6.89 (0.01)	0.035
	PL	2555.19 ± 361.5	2409.33 ± 617.6				0.139
RMR/BM (kcal/day/kg)	WBV	28.52 ± 3.16	31.15 ± 4.81	3.02 (0.09)	0.15 (0.69)	5.82 (0.02)	0.053
	PL	33.57 ± 4.37	31.45 ± 6.57				0.169
RMR/BSA (kcal/day/m^2^)	WBV	1112.94 ± 124.6	1217.88 ± 202.0	2.37 (0.13)	0.33 (056)	5.01 (0.03)	0.052
	PL	1302.44 ± 168.4	1233.40 ± 294.9				0.256
CHO (g/day)	WBV	330.88 ± 98.1	407.13 ± 111.9	0.0001 (0.99)	6.91 (0.01)	0.45 (0.50)	0.024
	PL	341.19 ± 127.9	391.27 ± 125.1				0.184
FAT (g/day)	WBV	71.75 ± 39.96	59.38 ± 41.83	2.35 (0.13)	4.68 (0.0)	1.05 (0.31)	0.459
	PL	111.19 ± 57.78	74.47 ± 77.91				0.034
PRO (g/day)	WBV	23.69 ± 3.14	26.00 ± 4.79	3.08 (0.09)	0.05 (0.81)	7.14 (0.01)	0.045
	PL	28.69 ± 4.06	26.73 ± 7.24				0.100
EE_CHO (kcal/h)	WBV	56.56 ± 16.72	69.81 ± 19.18	0.00 (0.99)	7.09 (0.01)	0.49 (0.48)	0.022
	PL	58.50 ± 21.87	67.07 ± 21.46				0.182
EE_FAT (kcal/h)	WBV	28.00 ± 15.44	22.94 ± 16.27	1.92 (0.1)	4.54 (0.04)	0.76 (0.39)	0.372
	PL	41.69 ± 22.11	28.73 ± 30.17				0.045
EE_PRO (kcal/h)	WBV	4.19 ± 0.66	4.44 ± 0.89	2.83 (0.10)	0.96 (0.33)	7.35 (0.01)	0.223
	PL	5.06 ± 0.68	4.53 ± 1.25				0.015

*VO*_*2*_ oxygen uptake, *VCO*_*2*_ carbon dioxide production, *RQ* respiratory quotient, *RNR* resting metabolic rate, *BM* body mass, *BSA* body surface area, *CHO* carbohydrates, *PRO* proteins, *EE* energy expenditure, *WBV* whole-body vibration, *PL* placebo.

## Discussion

To the best of our knowledge, this is the first study to evaluate the effects of a series of WBV on resting metabolism. A previous study^17^, which only examined the effect of a single WBV on RMR, showed that RMR was increased during WBV and the effect disappeared after WBV session. This study showed that there was a significant increase in RMR in young men after just two weeks of WBV intervention.

Resting metabolism is affected by age, physical activity, and lean body mass^3^. Long-term effects of physical activity result in increases in RMR due to increases in lean muscle mass^3^ and lean body mass correlates highly with RMR^23^. Many other factors: anxiety, diurnal variation, the thermic effect of food, elevated post-exercise oxygen consumption, stimulants, and pharmaceuticals can also affect the resting metabolic rate^24^. In our study, both age, physical activity, and lean body mass were similar in both groups, so it is likely that they also had similar effects on metabolism in both groups. The participants in both groups also consumed similar amounts of carbohydrates, proteins and fats. The study’s inclusion criteria also allowed for ineligibility of stimulant users and pharmaceuticals. The participants enrolled the study were rested (after an overnight rest) and avoided physical exertion for three days prior to RMR measurement. This allowed us to control most of the factors that could affect RMR. Haugen et al.^24^, also showed that repeated morning and evening measurements of RMR are stable and highly correlated and day-to-day measurements of RMR are not significantly different. This indicates that RMR measurement is highly repeatable.

In this study, by applying WBV in the prone position, it was possible, on the one hand, to deactivate postural muscles and, on the other hand, to apply additional local vibration, to stimulate directly the abdominal cavity. Local vibration, could affect the function of the gastrointestinal tract and thus, indirectly, the thermic effect of food (TEF) i.e. the energy cost of chewing, swallowing, digesting, absorbing and storing food. TEF is a significant part of energy expenditure and is influenced by, among other things, the caloric content of the diet, eating habits, physical activity and sympathetic nervous system activity^25^. It has been shown that sympathetic stimulation in the upper gastrointestinal tract may increase metabolic rate up to 15.6%^26^. Previous paper has shown that WBV can be an effective method in improving gastrointestinal function. Two weeks of low-intensity WBV was an effective therapy for reducing symptom severity in patients with chronic functional constipation^27^. For this reason, vibrating capsules have begun to be used in patients with constipation. Vibrating capsule may improve constipation by augmenting the physiological effects of waking and meals on bowel movements and circadian rhythm^28^. Vibrating capsule can promote defecation, as well as ameliorating symptoms and improving the quality of life in patients with functional constipation with sustained efficacy^29^. The applied local abdominal vibration was a specific abdominal vibro-massage. Abdominal massage can stimulate the parasympathetic activity and thus the gastrointestinal response^30^. It increases peristaltic movements and accelerates the passage of food through the gastrointestinal tract^31,32^. Our participants did not declare constipation or have a medical interview in this direction, nor did they report functional changes in the gastrointestinal tract after intervention, this applied local vibration, targeting the abdominal cavity, could significantly affect gastrointestinal tract function and improve its function, thus affecting TEF.

The study used WBV in combination with local vibration. Although local vibration may have affected TEF, the observed metabolic effect may also be due to the effects of WBV or the combination of local vibration and WBV. Increased metabolism during WBV may also be the result of changes in circulation because the peripheral vascular system is sensitive to vibration stimulation^33^. In a previous study^8^, an increase in mean blood flow was observed during WBV. Whole-body vibration also increases muscle activity by inducing a tonic vibration reflex in the muscles^34^. RMR is mostly dependent on the amount of metabolically active tissue in an individual (mainly muscle mass)^35^. Thus, both increasing muscle activity and improving tissue blood flow can also affect metabolic rate.

## Conclusion

The results of our study indicate that after just 2 weeks of using WBV in combination with local vibration applied to the abdominal area, resting metabolism increased significantly. This indicates that this type of vibration can be an addition to obesity therapy, in which WBV can, among other beneficial metabolic effects, increase RMR and thus total energy expenditure.

### Strength and limitation of the study

The strength of this study is the rigorous methodology and research methods used. Our intervention lasted 2 weeks, and after that time we observed an increase in RMR in men. Perhaps the results would have been different after a different intervention period. The reported effect applies only to vibration with the described characteristics and body position. Only young healthy men without overweight or obesity participated in this study. Further studies should also be conducted in women and those with obesity or other metabolic disorders to confirm the reported effect.

## Data Availability

The data presented in this study are available on request from the corresponding author.
